# Esophageal Perforation and EVAC in Pediatric Patients: A Case Series of Four Children

**DOI:** 10.3389/fped.2021.727472

**Published:** 2021-08-06

**Authors:** Laura Antonia Ritz, Mohammad Samer Hajji, Tobias Schwerd, Sibylle Koletzko, Dietrich von Schweinitz, Eberhard Lurz, Jochen Hubertus

**Affiliations:** ^1^Department of Pediatric Surgery, Dr. von Hauner Children's Hospital, Ludwig-Maximilian-University of Munich, Munich, Germany; ^2^Department of Pediatric Gastroenterology, Dr. von Hauner Children's Hospital, Ludwig-Maximilian-University of Munich, Munich, Germany

**Keywords:** esophageal perforation, VAC, vacuum therapy, children, pediatric patients

## Abstract

**Introduction:** In pediatric patients, esophageal perforation (EP) is rare but associated with significant morbidity and mortality rates of up to 20–30%. In addition to standard treatment options, endoscopic esophageal vacuum-assisted closure (EVAC) therapy has shown promising results, especially in adult patients. Thus far, the only data on technical success and effectiveness of EVAC in pediatric patients were published in 2018 by Manfredi et al. at Boston Children's Hospital. The sparse data on EVAC in children indicates that this promising technique has been barely utilized in pediatric patients. More data are needed to evaluate efficacy and outcomes of this technique in pediatric patients.

**Method:** We reviewed five cases of therapy using EVAC, ArgyleTM Replogle Suction Catheter (RSC), or both on pediatric patients with EP in our institution between October 2018 and April 2020.

**Results:** Five patients with EP (median 3.4 years; 2 males) were treated with EVAC, RSC, or a combination. Complete closure of EP was not achieved after EVAC alone, though patients' health stabilized and inflammation and size of EP decreased after EVAC. Four patients then were treated with RSC until the EP healed. One patient needed surgery as the recurrent fistula did not heal sufficiently after 3 weeks of EVAC therapy. Two patients developed stenosis and were successfully treated with dilatations. One patient treated with RSC alone showed persistent EP after 5 weeks.

**Conclusion:** EVAC in pediatric patients is technically feasible and a promising method to treat EP, regardless of the underlying cause. EVAC therapy can be terminated as soon as local inflammation and C-reactive protein levels decrease, even if the mucosa is not healed completely at that time. A promising subsequent treatment is RSC. An earlier switch to RSC can substantially reduce the need of anesthesia during subsequent treatments. Our findings indicate that EVAC is more effective than RSC alone. In some cases, EVAC can be used to improve the tissues condition in preparation for a re-do surgery. At 1 year after therapy, all but one patient demonstrated sufficient weight gain. Further prospective studies with a larger cohort are required to confirm our observations from this small case series.

## Introduction

In pediatric patients, esophageal perforation (EP) is rare but associated with high morbidity rates of up to 20–30% (1). The most common cause for EP (75%) in children is dilatation of preexisting stenosis (2). The overall risk of perforation after dilatation is only 0.6% for all types of stenosis (3) but this risk increases up to 3.4–18% for congenital stenoses of the esophagus (4, 5). Other reasons for perforation can be foreign body ingestion or anastomotic leak after esophageal anastomosis (1, 2). In neonates, nasogastric tube insertions and attempted endotracheal intubation are main sources of EP (6). Interrupted continuity of the esophageal wall can cause saliva, bacteria, and digestive enzymes to migrate into the mediastinum, which can lead to empyema, abscess formation, and mediastinitis and potentially progress to sepsis and necrosis of pulmonary tissue (7).

The treatment for EP used to be primary repair (8–10) or stenting (11) for both pediatric and adult patients. Whereas, EP in adults commonly occurs with underlying pathology (1), thus favoring invasive approaches, EP in children mostly occurs in vital tissue with greater propensity to heal (1, 2), in which case non-operative treatments (e.g., parenteral nutrition and broad-spectrum antibiotics) may be preferred, as described by Van der Zee et al. (12).

Over the last several years, non-operative techniques have been introduced in adult patients. Among these, endoscopic esophageal vacuum-assisted closure (EVAC) therapy has shown promising results. This well-established technique originated as a treatment for infected wounds, burns, and ulcers (13–18). In 2011, Loske et al. (19) and Schorsch et al. (20) modified it for intraluminal use in adult patients with EP. Multiple studies have verified the success of EVAC (19–26) for this purpose and have shown that it is more effective than stents (27).

The only data on the technical success and effectiveness of EVAC among pediatric patients were published in 2018 by Manfredi et al. (28) from Boston Children's Hospital (29). This promising technique therefore needs further investigation in pediatric patients. We aim to describe our experience using a therapy combining EVAC with an Argyle^TM^ Replogle Suction Catheter (RSC) in pediatric patients with EP.

In our hospital, stents are not used in the treatment of EP. The therapy used to be non-operative with parenteral nutrition and broad-spectrum antibiotics and was then extended by the additional use of EVAC.

In our patients EVAC was technically possible and effective. No patient experienced complete closure of EP after EVAC alone, though patients were subsequently treated with RSC until restitutio ad integrum.

## Materials and Methods

We performed an institutional review on all patients aged ≤18 years with EP who were treated with EVAC or RSC between October 2018 and April 2020. Indication for EVAC therapy was a radiologically and endoscopically proven perforation of the esophagus with saliva leaking into the mediastinum. Patients aged >18 years or those with perforation of the esophagus or fistulation into the trachea or bronchi were excluded.

The EVAC sponge was placed using a size-adapted gastroscope and inserted antegrade. Correct position was confirmed by a retrograde endoscopy over a gastrostomy, in analogy to Manfredi et al. (28). An Eso-SPONGE®-system (Braun, Melsungen, Germany) was cut to size according to the patient's size. If an extraluminal cavity was present, the sponge was initially placed in the cavity and retracted with every session. If there was no extraluminal cavity, the sponge was placed in the esophagus, overlapping the EP on both sides. The position of the sponge was secured by a holding thread which was passed out and secured over the gastrostomy. The tube was connected to a Medela Thopaz+® Digital Chest Drainage and Monitoring System (Medela, Baar, Switzerland). Negative pressure of −100 cm H_2_O was applied. Blood inflammation levels, mainly C-reactive protein (CrP), were determined before and during treatment. Antibiotics were given in a weight-adjusted dosage according to the antibiotic susceptibility test results.

The correct position was confirmed using a flexible endoscope. In the first two patients, the sponge was initially changed every 3 days. To minimize the number of anesthesia, intervals were lengthened to 5 days for these and all patients thereafter. Prior to removal, the sponge was flushed with 10–20 ml of NaCl 0.9% to avoid mucosal defects. Removal was performed under the guidance of the holding thread. After several intervals of EVAC, the sponge was replaced by an ArgyleTM® Replogle Suction Catheter (RSC). The RSC was connected to a Medela Thopaz+® Digital Chest Drainage and Monitoring System using a negative pressure of −100 cm H_2_O. The RSC was inserted endoscopically and placed at the level of the perforation. The correct position was checked radiologically. Patient 5 was treated with RSC alone.

The primary outcome was technical feasibility of EVAC in pediatric patients. The secondary outcome was the impact of an earlier switch to RSC on number of anesthesia. Finally, we assessed efficacy of RSC treatment alone and the medium-term outcome at 1 year after EVAC or RSC. Results are presented in a descriptive manner due to the size of our cohort.

Since this is a retrospective single-center study, data was collected prior to the study at our institution in the progress of treating the patients. As an inhouse-research, no approval of the ethics committee is needed.

## Results

Between October 2018 and April 2020, five pediatric patients (aged 7 months to 11.3 years, median 3.4 years; 2 males) with EP were treated with EVAC and/or RSC at our hospital. Patients 1 and 2 were born with esophageal atresia Gross type D and developed anastomotic leakage after failed primary repair and re-do surgery. Patient 3 was transferred to our hospital after iatrogenic EP post-dilatation of a congenital distal esophageal stenosis. Patient 4 suffered from anastomotic leakage after gastric transposition due to caustic injury at the age of 4 years. Gastric transposition was performed because of recurrent fistula and refractory esophageal stenosis despite multiple dilatations. Patient 5 was born with biliary atresia and treated with a Kasai procedure (heopatoportoenterostomy) and liver transplantation. Over time, the patient developed esophageal varices. After massive bleeding with neurologic consequences, a nasogastric tube was blindly placed causing EP. Later, an esophagogastric fistula was detected endoscopically. Table 1 summarizes the clinical data.

**Table 1:** Patients treated with endoscopic esophageal vacuum-assisted closure (EVAC) between 2018 and 2020.

**Patient**	**1**	**2**	**3**	**4**	**5**
Age /month	41	7	30	136	24
Diagnosis	EA^1^ Gross D	EA Gross D	CES^2^	Caustic injury	BA^3^, liver Tx^4^
Reason for treatment	AL^5^	RF^6^	IP^7^	AL	EGF^8^
Position of EP^9^	T^10^3	T5/6	T11	T6/7	T11/12
**EP distance to dental arch**					
Lenght of EVAC^11^	30	21	11	12	24^*^
Sessions	8	6	2	3	4
Subsequent therapy	RSC^12^	Surgery	RSC	RSC	RSC
Days of treatment	45	21	26	23	38
Diet after 1 year	PEG^13^/SF^14^	Normal diet	Normal diet	Normal diet	PEG

*For Patients 1 and 2, the sponge was changed every 3 days. For the other patients, it was changed every 5 to 6 days, and duration of EVAC therapy was reduced. The Replogle Suction Catheter (RSC) was continued until the esophageal perforation (EP) healed. Patient 5 was treated with RSC alone and had a persistent fistula at 5 weeks after treatment*.

1 *EA, Esophageal atresia;*

2 *CES, Congenital esophageal stenosis;*

3 *BA, Biliary atresia;*

4 *Tx, Transplantation;*

5 *AL, Anastomotic leak;*

6 *RF, Recurrent fistula;*

7 *IP, Iatrogenic perforation;*

8 *EGF, Esophagogastric fistula;*

9 *EP, Esophageal perforation;*

10 *Tx, Thoracic vertebral body;*

11 *EVAC, Endoluminal vacuum-assisted closure therapy;*

12 *RSC, Replogle suction catheter;*

13 *PEG, percutaneous endoscopic gastrostomy;*

14 *SF, Soft food;*

* *RSC only*.

**Figure 1:**
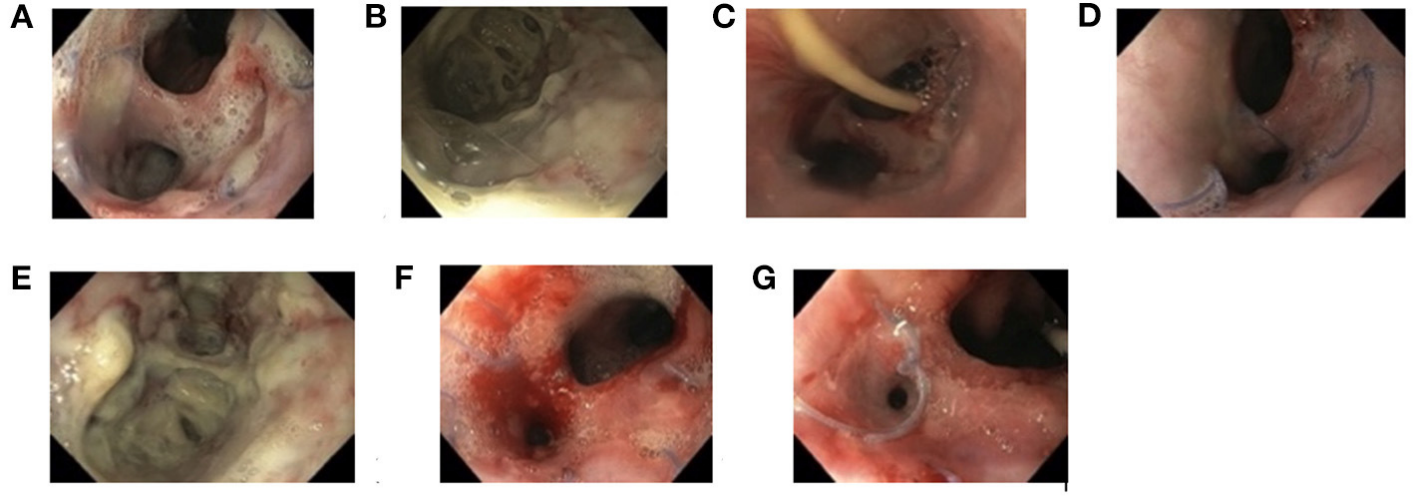
Documentation of endoscopic findings during endoscopic esophageal vacuum-assisted closure (EVAC) and Replogle Suction Catheter (RSC) therapy (Patient 4). First impression of anastomotic insufficiency forming a second lumina into the mediastinum **(A)**, shown more closely **(B)**. Esophageal perforation after 4 days **(C)**, 8 days **(D)**, and 12 days **(E)** of EVAC therapy. After day 8, EVAC was replaced by an RSC **(F)**. Fully healed esophageal perforation 10 days after RSC **(G)**.

**Figure 2:**
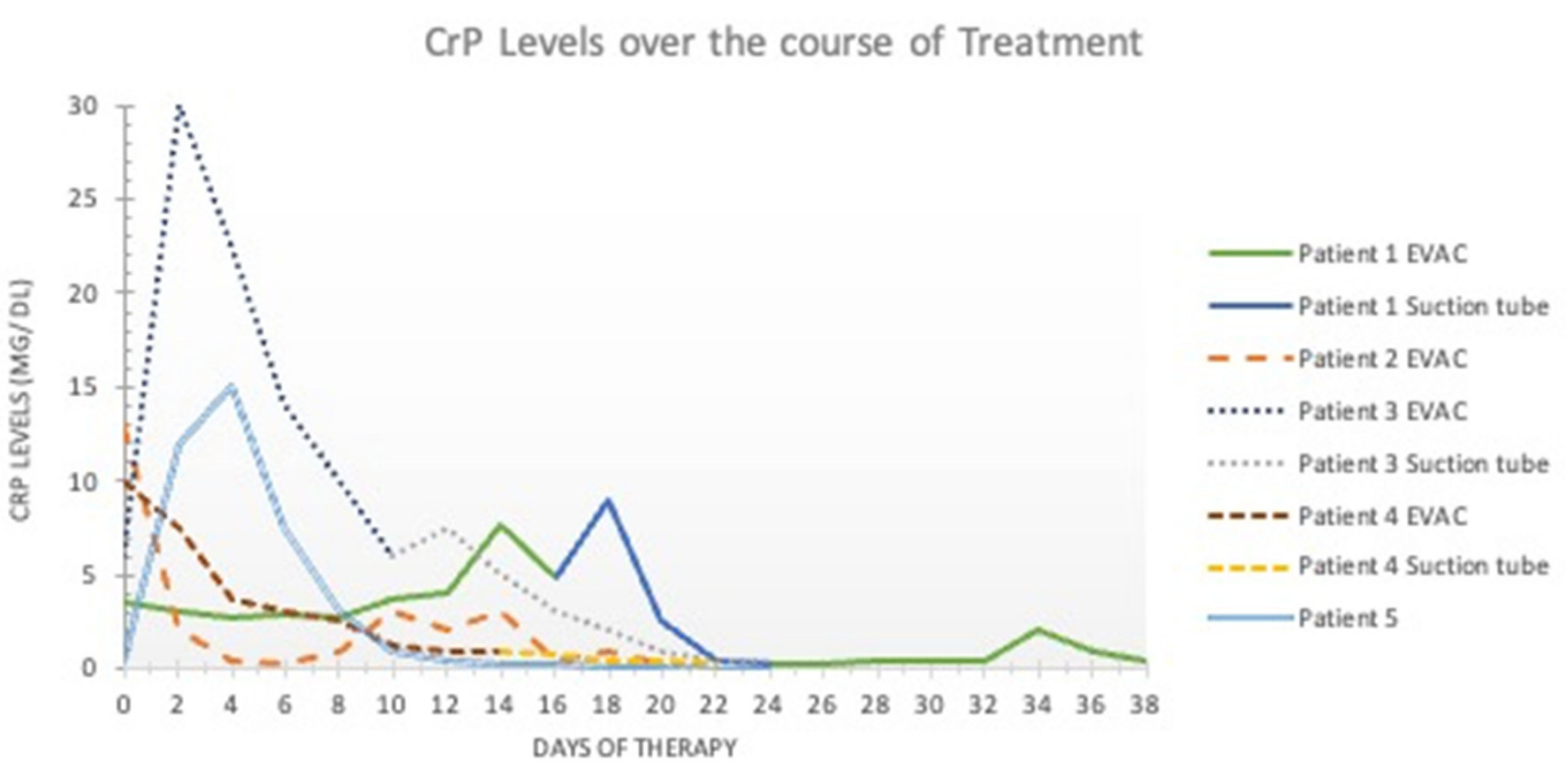
C-reactive protein levels over time. The x- axis shows the duration of esophageal perforation therapy (in days). The y-axis shows the C-reactive protein levels in milligrams per deciliter (mg/dL). Values < 0.5 mg/dL are considered normal and without inflammation. EVAC, endoscopic esophageal vacuum-assisted closure; CRP, C-reactive protein.

In Patients 1 and 2, the sponge was initially changed every 3 days, and EVAC therapy was administered for 21 and 30 days, respectively. In Patients 3 and 4, EVAC therapy was shortened to 11 and 12 days, respectively. None of these 4 patients experienced complete EP closure after EVAC alone (Figure 1). However, their health stabilized, EP size decreased, and initially high CrP-levels (as a marker of inflammation) normalized (Figure 2). All four patients were subsequently treated with RSC until their EP healed.

With increasing experience in EVAC therapy, time between EVAC sessions was prolonged from 3 to 5 to 6 days to minimize anesthesia. Learning from the experience of Patient 1 and 2, the RSC was placed as soon as CrP-levels began falling and when mucosal inflammation and size of EP macroscopically decreased (Figure 1). All patients were treated with antibiotics according to antibiotic susceptibility test results until the EP healed.

In Patient 2, recurrent fistula did not heal after 3 weeks (six sessions) of EVAC therapy, thus requiring surgery. But EVAC therapy reduced local inflammation in this patient, thereby reducing the risk of major or fatal complications during surgery. No other patient required surgery. Patient 1 and 3 developed stenosis after EVAC, and both were successfully treated with dilatations.

Based on shortening the duration of EVAC therapy from 21–30 days to 11–12 days, it was decided to completely dispense EVAC for Patient 5. The patient's EP was instead treated using an RSC and broad-spectrum antibiotics only. Despite normalizing CrP levels, the EP persisted after 5 weeks. The patient continues to be fed via gastrostomy.

**Figure 3:**
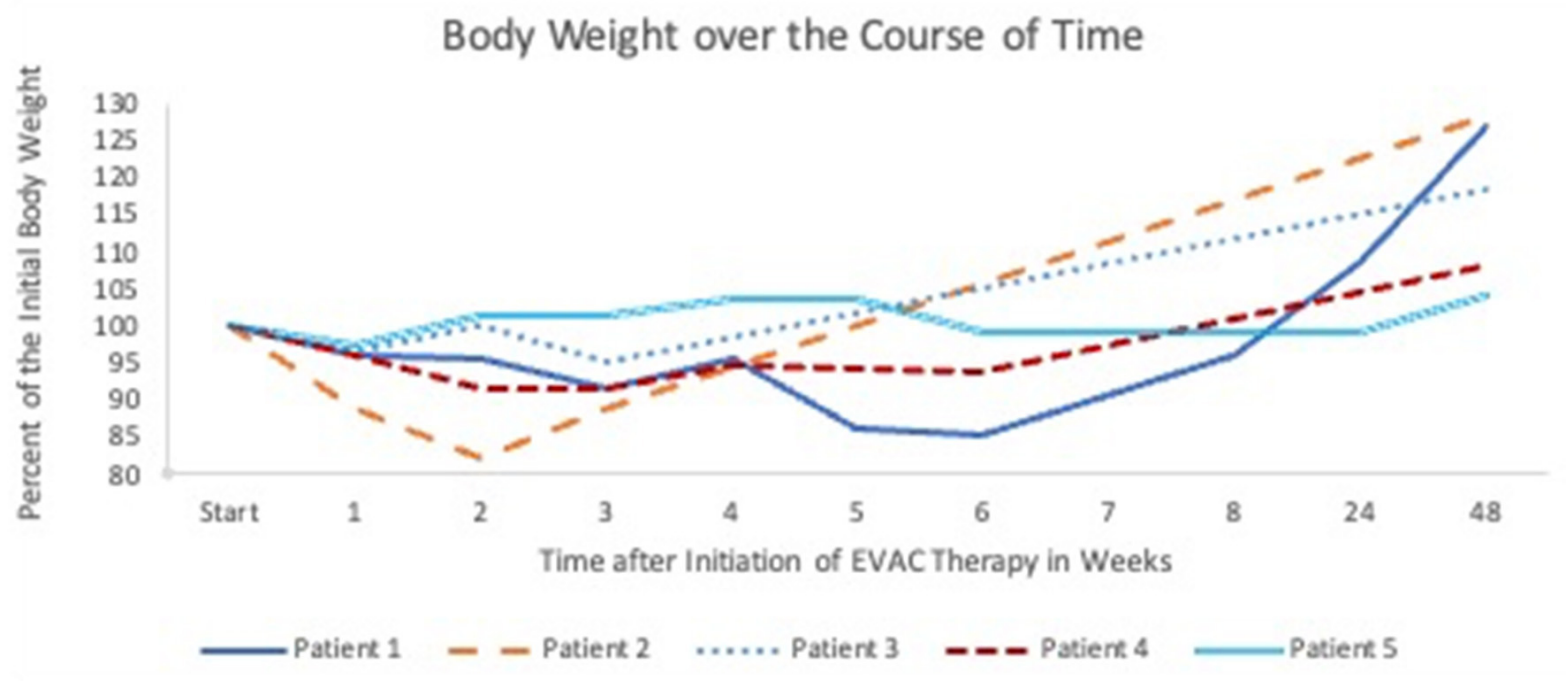
This graph shows the course of body weight over the 1 year after treatment.

Oral feeding is not possible during EVAC therapy. Prior to the EP, Patients 1 and 4 were already partially fed via gastrostomy. Patient 2 received a gastrostomy with the first placement of EVAC. Whether patients were enterally fed through a gastrostomy or with parenteral nutrition alone during the 6 week study period, weight loss, in general, was <10% (Figure 3). Patient 2 experienced rapid weight loss due to the persistent EP, and the patient's weight returned to normal within 4 weeks after surgery. Enteral nutrition was initiated in all patients after fluoroscopy confirmed a closed EP. All but Patient 5 gained sufficient weight (30) at 1 year after EVAC therapy started.

## Discussion

Vacuum-assisted closure is a well-established technique of applying sub-atmospheric pressure to treat infected wounds, burns, or ulcers (13–18). The pressure is applied continuously or in alternating 5-min cycles with 2-min pauses (13). This technique helps remove bacteria and excess fluids to improve blood flow and formation of granulation tissue (13, 17). Endoluminal vacuum-assisted closure was first described by Weidenhagen et al. o repair anastomotic leakage after anorectal surgery (31). In 2011, Loske et al. adapted this method for use in the upper gastro-intestinal tract (19). Since then, therapy of EP in adult patients has shifted from surgical repair (8, 10) or stenting (11) to conservative treatment (32).

The overall healing rate of EP treated with EVAC is reportedly between 70 and 100% (19–21, 23, 33–35), which is superior to that for stenting (28). Complete closure was not achieved in a single patient with EVAC alone. But the combination of EVAC and RSC was successful in 75%. Maybe the sponge was still too big, even after size adaption. Thus, the vulnerable tissue in children might be affected by the pressure of the sponge. Therefore, complete healing was only achieved by RSC as subsequent therapy.

All patients showed significant reduction in local and systematic inflammation, shortly after insertion of EVAC. Therefore, EVAC was helpful even in the one patient who needed re-do surgery in the end. In this case, local inflammation was reduced and operative field was well-prepared for the surgery. The results indicate that in some cases, EVAC facilitates improved outcomes in subsequent surgery even if the EVAC itself does not fully resolve the EP.

Finally, our results of a very small cohort showed a success rate of 75% for EVAC and RSC, which is similar to results of pediatric patients described by Manfredi et al. (28).

After treating the first two patients we learned that increasing the interval between EVAC sessions to 5 days was well tolerated by the patients, thus reducing the number of anesthesia. This approach was similarly described by Schorsch et al. (20) and Bludau et al. (34, 35).

Mean duration of EVAC therapy described in the first study was 17 days for adult patients (19). Later studies reported have a median duration of 11 to 12 days (20, 34, 35). In our cohort of four pediatric patients, the median EVAC duration was 18 days. When local inflammation and CrP levels decreased sufficiently in these patients, EVAC was replaced by RSC until the mucosa completely healed. The duration of EVAC therapy was shortened from initially 30 to 11 days in the last patient. RSC was installed earlier with good results. The space consuming effect of the sponge might have been a hindrance to a complete closure. Nevertheless, the sponge seems to be important for healing, as a complete omission of EVAC, as in Patient 5, did not yield a satisfactory result. Although only a single patient, this case nevertheless emphasizes the benefit EVAC therapy in treating EP.

The prolonged EVAC intervals and earlier use of RSC reduced anesthesia sessions significantly from 8 to 2 days.

All 5 patients were treated with antibiotics until the EP healed. As shown in the graph, it is worth assessing whether treatment with antibiotics should be terminated earlier due to rapidly falling CrP levels. EVAC was technically possible in four patients. Initially, removal was difficult due to the sponge being too dry. Flushing the sponge with 10–20 ml NaCl 0.9% solved the problem.

At our hospital, stents have not been used, therefore we have only little experience in this field. After our opinion, stents are not a save option in children as they tend to dislocate (36) and bear the risk of damaging the vulnerable tissue in children.

## Conclusion

EVAC in pediatric patients is a technically feasible and promising method to treat EP, regardless of etiology. EVAC therapy can be terminated as soon as local inflammation and CrP levels decrease sufficiently, even if the mucosa is not yet healed. A promising subsequent therapy is use of an RSC. An early switch can decrease anesthesia time drastically. Overall, EVAC appeared to be more effective than RSC alone. We also observed that EVAC improved the tissue condition in preparation for re-do surgery. At 1 year after therapy, all patients but one gained sufficient weight. Further prospective studies with a larger cohort are required to confirm our experience from this small case series.

## Data Availability Statement

The original contributions presented in the study are included in the article/supplementary material, further inquiries can be directed to the corresponding author/s.

## Author Contributions

All authors listed have made a substantial, direct and intellectual contribution to the work, and approved it for publication.

## Conflict of Interest

The authors declare that the research was conducted in the absence of any commercial or financial relationships that could be construed as a potential conflict of interest.

## Publisher's Note

All claims expressed in this article are solely those of the authors and do not necessarily represent those of their affiliated organizations, or those of the publisher, the editors and the reviewers. Any product that may be evaluated in this article, or claim that may be made by its manufacturer, is not guaranteed or endorsed by the publisher.
